# Experience with Pressure Anesthesia

**Published:** 1900-02-15

**Authors:** E. M. Cook

**Affiliations:** Toledo, Ohio


### EXPERIENCE WITH PRESSURE ANESTHESIA.

BY E. M. COOK, D.D.S., TOLEDO, OHIO.

I had a case last year which was very interesting to me and might be instructive to those who have not had much experience with the pressure anesthesia method of removing pulps. The patient came to me with a highly-inflamed pulp and a great deal of pericementitis; the tooth was long and it was impossible to put the least amount of pressure upon it. By removing as carefully as possible an old filling that covered the pulp, I was enabled to introduce a very strong alcoholic solution of cocain, but the slightest pressure of the cotton caused such severe pain that the patient could hardly endure it. After working carefully and making slight pressure with cotton, I was then enabled to introduce the soft rubber over the cotton and began by making a very light pressure, and after a few moments I was enable to increase the pressure and, as the operation continued, the sensitiveness of the tooth decreased until I was able to put all the pressure upon the tooth that was necessary to drive the cocain into the pulp completely and I removed the pulp, which was in a highly-sensitive condition, without any or very little pain to the patient; after thoroughly drying I filled it immediately, and there was no recurrence of soreness after the operation. I have since last February had but one case of the removal of a pulp from a root canal that has not given me perfect satisfaction by the use of pressure anesthesia.

